# Assessment of three DNA extraction kits for the absolute quantification of strongyle nematode eggs in faecal samples

**DOI:** 10.1186/s13028-022-00624-3

**Published:** 2022-02-09

**Authors:** Niclas Högberg, Paulius Baltrušis, Nizar Enweji, Johan Höglund

**Affiliations:** Parasitology Unit, Department of Biomedical Sciences and Veterinary Public Health, Swedish University of Agricultural Sciences, Box 7036, 750 07 Uppsala, Sweden

**Keywords:** Diagnostics, DNA isolation, *Haemonchus*, Nematode eggs, Parasite, PCR, Sheep

## Abstract

**Background:**

*Haemonchus contortus* is one of the most pathogenic gastrointestinal nematodes of small ruminants. The current diagnostic approach for the detection of this species relies on coproscopic methods, which both have low sensitivity and are time consuming. Methods employing detection through DNA amplification, such as droplet digital polymerase chain reaction (ddPCR), offer an advantageous approach to the diagnosis of *H. contortus*. However, DNA extraction protocols need to be constantly updated for the optimal retrieval of diagnostically usable template. Here, we describe the evaluation of three genomic DNA extraction kits for the detection and quantification of *H. contortus* ITS2 amplicon DNA from faecal samples, using droplet digital PCR.

**Results:**

DNA samples, extracted from faecal material with the Nucleospin DNA Stool kit, produced the highest amounts of ITS2 amplicon copies and had the lowest coefficient of variation across different dilutions and sample types (fresh or frozen) out of the tested kits (Nucleospin DNA Stool, E.Z.N.A.^®^ Stool DNA Kit and QIAamp Fast DNA Stool Mini Kit). Furthermore, the protocol of this kit has the fewest number of steps and the price of DNA extraction per sample is reasonable (2.77 €).

**Conclusions:**

The Nucleospin DNA Stool kit is an attractive option for the detection and quantification of *H. contortus* DNA in faecal samples of small ruminants in a diagnostic setting.

## Background

Gastrointestinal nematodes (GIN) present substantial problems in the livestock sector worldwide [1]. *Haemonchus contortus* is one of the most pathogenic and commonly encountered parasitic GIN. It largely affects both the animal health and farm productivity and profitability [2]. Furthermore, anthelmintic resistance (AR) has been reported from most sheep rearing-countries in the world [3–6]. It is therefore necessary to maintain vigilance for the presence of this species on farms producing sheep and goats, especially in light of increasing number of reports of anthelmintic resistance [7].

Current field diagnostics rely on coproscopic methods and the subsequent visual identification of the infective third stage larvae (L3) to confirm the presence (or absence) of *H. contortus* on any particular farm. However, this approach not only requires expertise in the form of trained technical staff as well as being laborious and time consuming as the culturing of larvae for more than a week is usually required for the identification of morphologically similar strongylid eggs [8]. It has also been demonstrated that the culturing conditions for nematode eggs may impose a bias towards the species composition in the larval cultures [8]. For some applications, such as the declaration of freedom from parasites when purchasing/selling animals, quarantine and treatment evaluation as an indication of treatment failure, there is a need to establish automated and sensitive methods that are precise, less time consuming and labour intensive. Molecular approaches, based on template amplification, offer superior sensitivity, precision and more rapid parasite identification, in comparison to the more traditional culturing methods [9, 10]. However, such methods have only rarely been applied directly to parasite eggs contained in faecal samples, without the prior concentration and harvest of the eggs from faeces by flotation [11–13].

As is the case with all polymerase chain reaction (PCR) based diagnostics, the key step is the acquisition of a sufficient amount and quality of template genomic DNA (gDNA). To date, few studies have systematically explored the suitability of various commercially available DNA extraction kits in order to obtain genomic DNA from parasites of veterinary importance for subsequent analyses [11, 14, 15]. Nevertheless, although a variety of commercial kits for DNA extraction are available on the market, comparatively little is known about the efficacy of each on the quantitative molecular detection of *H. contortus* in faecal samples containing the parasite’s eggs. In addition, each nematode species possesses unique eggshell compositions making the DNA extraction step not only crucial to the outcome of the parasite detection but also subject to variation in efficiency, based on the chosen approach. Therefore, comparative studies evaluating DNA extraction from nematode eggs, subjected to different conditions, are necessary in order to find optimized and standardized protocols for DNA acquisition.

Droplet digital PCR (ddPCR) is a versatile and robust platform designed for selective amplification and probe-driven detection of short (typically 50–250 bp long) amplicons. We have previously successfully implemented ddPCR to quantify the presence of strongyle nematodes in larval cultures, derived from livestock samples, in order to distinguish between the major parasite genera with high precision [16, 17]. Furthermore, assays, based on ddPCR have been found to exhibit superior sensitivity [18] as well as increased tolerance to inhibitors in comparison to more conventional PCR approaches [19]. Thus, ddPCR is an excellent tool for precise estimation of small quantities of amplicon DNA, such as those derived from faecal samples, containing a limited number of *H. contortus* eggs.

Herein, we describe a series of experiments evaluating the suitability of three commercial DNA extraction spin-column based kits (Nucleospin DNA Stool, E.Z.N.A.^®^ Stool DNA Kit and QIAamp Fast DNA Stool Mini Kit) for the subsequent molecular, quantitative detection of *H. contortus* amplicon DNA from either fresh or frozen faecal samples, using the previously reported ddPCR setup [16].

## Methods

### Sample preparations and experimental design

**Fig. 1:**
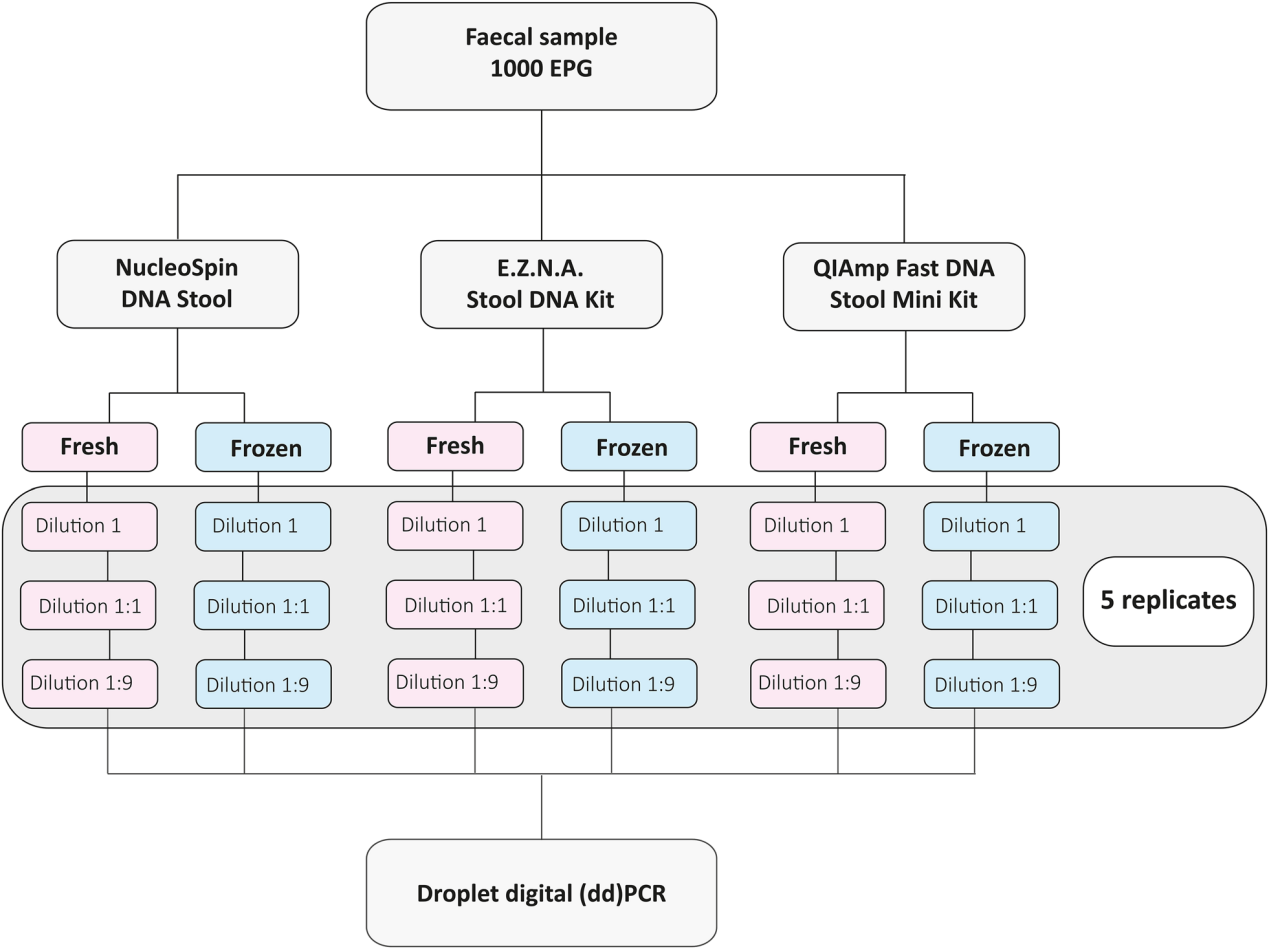
Flow chart illustrating the experimental design for evaluating three DNA extraction kits, using a single faecal egg sample, monospecifically infected with *Haemonchus contortus*. Each kit was evaluated with five replicates of fresh and frozen samples of three dilutions (1; 1:1 and 1:9). Droplet digital (dd)PCR was carried out implementing a previously described protocol for the quantification of ITS-2 region copies in strongyles

The material used in this study originated from a single, fresh faecal sample from a sheep monospecifically infected with *H. contortus*, and was obtained from a routine veterinary diagnostic laboratory. Faecal egg counts (FEC) were determined through a modified McMaster method, including sieving (aperture size of 160 μm) and centrifugation (425*g*) steps, using a slurry, containing 3 g of faeces and 42 mL of water. The samples were finally redispersed in a saturated NaCl solution (specific gravity 1.2), providing a minimum detection level of 50 strongyle eggs per gram (EPG) of faeces. The procedure was repeated three times and the mean EPG was calculated. The remaining faecal slurry, when in tap water, was transferred into a 15 mL sterile tube (Sarstedt, Nümbrecht, Germany) and kept at 4 °C. The following day DNA was extracted from the fresh material. The remaining faecal slurry was stored at − 20 °C for 3 months before DNA extraction. To test for the consistency of target DNA copy estimation by droplet digital PCR, replicated samples containing either undiluted (1) or one of two dilutions (1:1 and 1:9) of the faecal slurry were prepared before the initial DNA extraction step for every kit. Dilutions were done by mixing either equal portions of the faecal slurry and distilled water (1:1) or 1 part of the faecal slurry and 9 parts of distilled water (1:9). For each kit, dilution and storage condition, five replicates were prepared (with final volume in each being 220 μL) (Fig. 1).

### DNA extraction

DNA was extracted from each of five technical replicates for every dilution category of the initial stock, either fresh or frozen, with three different commercial kits: (1) NS = Nucleospin DNA Stool (Macherey-Nagel), (2) EZ = E.Z.N.A.^®^ Stool DNA Kit (Omega), and (3) QA = QIAmp Fast DNA Stool Mini Kit (Qiagen). The NS and EZ kits employ a bead beating step in order to achieve mechanical lysis which in our case was achieved using Precellys Evolution homogenizer (10,000 RPM, 5 cycles × 60 s with a 20 s pause in between each cycle), whereas the QA kit relies solely on the chemical lysis as the mode of obtaining gDNA from samples. The kits were chosen due to their relative popularity and more importantly—their immediate availability. The extraction protocols were carried out by closely following the guidelines issued by the manufacturers. For the NS kit, the protocol “Genomic DNA from stool samples” was carried out with initial lysis conducted at 70 °C for 5 min. The protocol “DNA Extraction and Purification from Stool for Pathogen Detection” was carried out when working with the EZ kit, with the initial lysis once again conducted at 70 °C for either 10 min or 13 min (if the sample is frozen). Finally, the protocol named “Isolation of DNA from Stool for Pathogen Detection” was carried out when evaluating the QA kit. Here the lysis was also done by heating the samples to 70 °C for 5 min and carefully following the instructions and notes issued by the manufacturers of the kit. To enable pipetting of the faecal slurry, pipet tips were cut prior to use.

### Droplet digital PCR

**Fig. 2:**
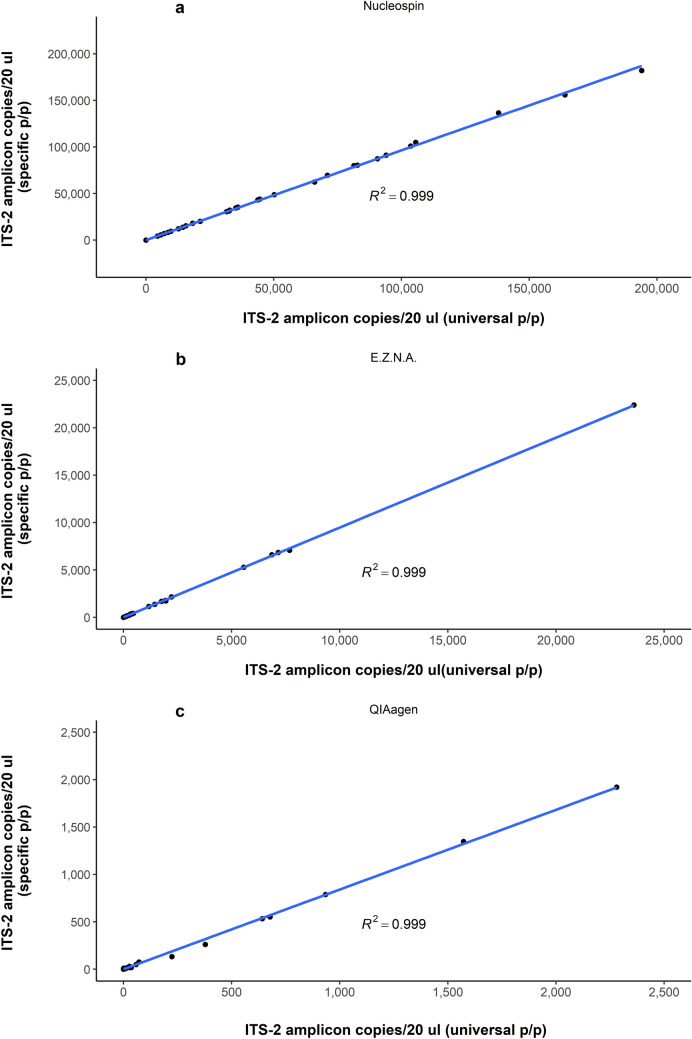
ITS2 amplicon copies/μL from a universal primer/probe compared to a *Haemonchus contortus* specific primer probe, from an initial faecal sample slurry, fresh or frozen, for three different dilutions (1; 1:1; 1:9) using: **a** NucleoSpin DNA Stool (n = 30), **b** E.Z.N.A. Stool DNA Kit (n = 30), and **c** QIAmp Fast DNA Stool Mini Kit (n = 30)

Droplet digital (dd)PCR was carried out using the extracted DNA samples as templates and implementing the previously described protocol for the quantification of ITS-2 region copies in major ovine strongyles [16]. Both *Haemonchus* specific as well as universal to all strongyles primer and probe sets were utilized to ensure a consistent quantification of the genus (Fig. 2). Reactions were assembled in 96-well plates (final volume 22 μL), following the guidelines issued by the manufacturer (BioRad). Droplets were generated and dispensed into a new 96-well plate using the automated droplet generator (QX200, BioRad). The new plate was heat sealed and transferred into a thermal cycler. The PCR conditions were as follows: a single cycle of 95 °C for 10 min, 40 cycles of 94 °C for 30 s. and then 57 °C for 1 min, followed by a single cycle of 98 °C for 10 min to deactivate the enzyme. After the amplification step, the plate containing the droplets was loaded into the droplet reader (QX200, BioRad) and further analysed using QuantaSoft (v1.7.4.0917) software, which generates DNA copy concentration measurements and error bars based on Poisson statistics (Droplet DigitalTM Applications guide http://www.bio-rad.com/webroot/web/pdf/lsr/literature/Bulletin_6407.pdf).

### Statistical analysis

The statistical analyses were performed using R studio (v. 1.2.5033). For every kit, the coefficient of variation (CV) (i.e. the standardised measure of the dispersion of data points around the mean) and dilution were analysed using the CV function in the sjstats package (version 0.18.1). The differences between the fresh and frozen samples and the dilutions were analysed with mixed models using LME function in the nlme package (version 3.1-152). Pairwise differences were estimated with ANOVA in the nlme package. Tukey’s pairwise comparisons were performed with the emmeans package (version 1.5.4). R-squared (R^2^) values were calculated using the lm function. The significance level was set to P < 0.05. All graphical illustrations were made using the ggplot2 package (version 3.3.3).

## Results

**Fig. 3:**
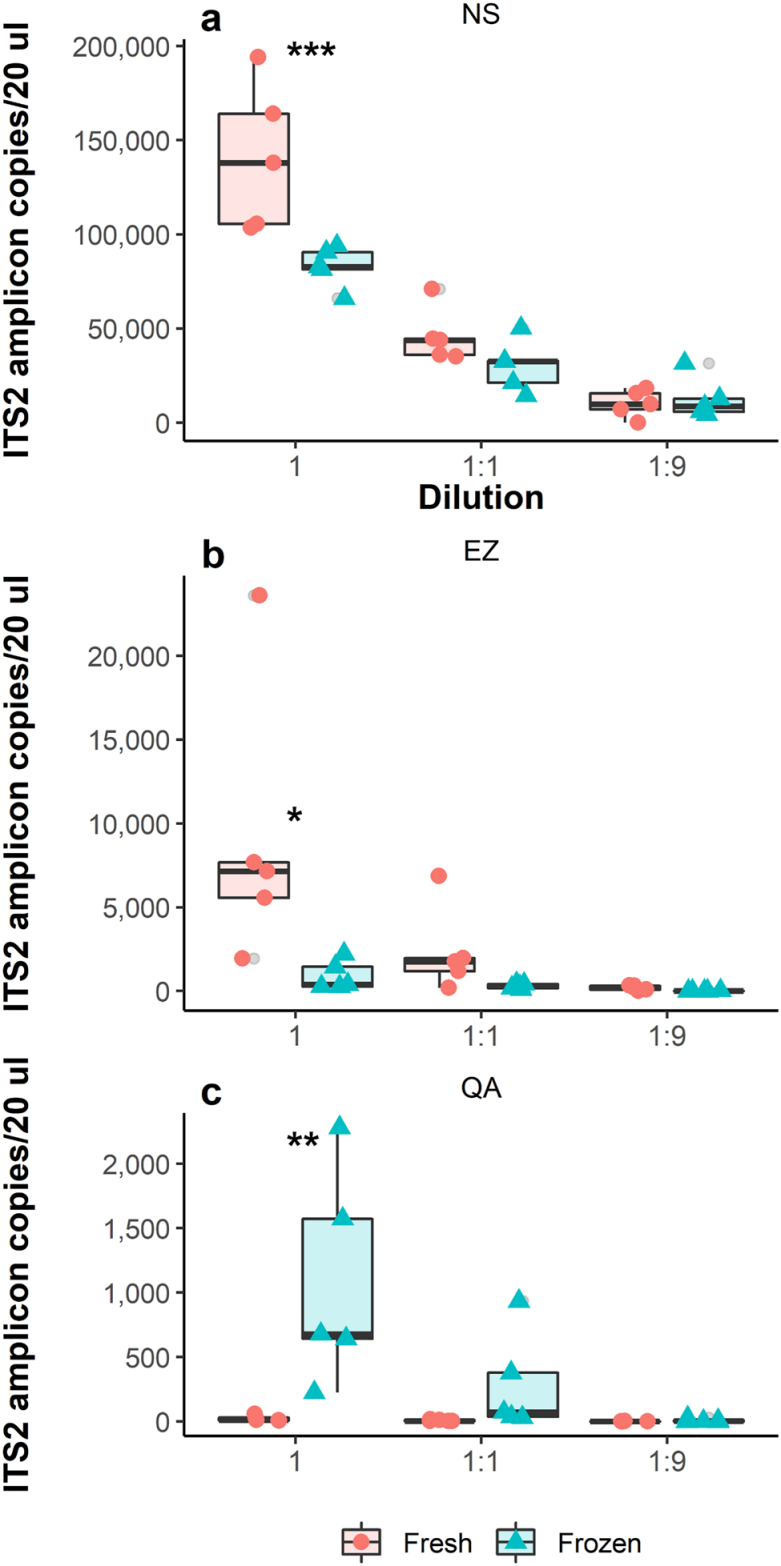
DNA yield (ITS2 amplicon copies/μL) from an initial faecal sample slurry, fresh or frozen, for three different dilutions (1; 1:1; 1:9) using: **a** NucleoSpin DNA Stool (n = 30), **b** E.Z.N.A. Stool DNA Kit (n = 30), and **c** QIAmp Fast DNA Stool Mini Kit (n = 30). Median values are indicated by the solid line within each box, with the box extending to the upper and lower quartile values. Outlier data points are indicated by grey dots. *P < 0.05, **P < 0.01, ***P < 0.001

We evaluated three commercial genomic DNA extraction kits for the detection and quantification of *H. contortus* ITS2 amplicon DNA faecal slurry samples, which were prepared either fresh or after they had been frozen at − 20 °C for 3 months. This in order to compare the performance of each kit using each five replicated samples prepared from the undiluted faecal slurry as well as when diluted 1:1 and 1:9 with distilled water. The mean FEC of the sample used in the study was determined to be 1083 ± 170 EPG. The *Haemonchus* ITS-2 amplicon concentration measurements were found to be different for all three tested kits irrespective of the dilution factor (Fig. 3). For the three kits, the differences in measurements between fresh and frozen samples were only significant when no dilutions were made (NS—P = 0.0009, EZ—P = 0.0146, QA—P = 0.0020). Contrary to both NS and EZ, undiluted and frozen samples extracted with QA yielded more ITS2 copies than fresh ones. The CV values (Table 1), as well as the highest overall number of ITS-2 copies, were obtained when the DNA was extracted using the NS kit from fresh faecal samples. Furthermore, the NS kit, overall, yielded more ITS2 DNA copies compared with both EZ (P < 0.0001) and QA (P < 0.0001), whereas no difference (P = 0.96) was observed between EZ and QA. Finally, the amplicon DNA concentrations in the diluted samples were lower than expected, compared to the undiluted, for all samples except one (NS frozen 1:9; Table 2).

**Table 1:** The coefficient of variation (CV) from an initial faecal sample slurry, fresh or frozen, for three different dilutions (1; 1:1; 1:9) for: (a) NucleoSpin DNA Stool, (b) E.Z.N.A. Stool DNA Kit, and (c) QIAmp Fast DNA Stool Mini Kit

Sample	n	Ratio faecal slurry:water	Coefficient of variation		
			NucleoSpin DNA Stool	E.Z.N.A. Stool DNA Kit	QIAmp Fast DNA Stool Mini Kit
Fresh	5	1	27.5	91.0	102.8
Fresh	5	1:1	31.6	108.8	100.9
Fresh	5	1:9	71.2	78.6	223.6
Frozen	5	1	13.1	96.5	77.1
Frozen	5	1:1	45.1	52.8	133.8
Frozen	5	1:9	87.1	181.3	158.8

**Table 2:** The arithmetic mean and standard deviation of DNA yield from an initial faecal slurry, fresh or frozen, for three different dilutions (1; 1:1; 1:9) for: (a) NucleoSpin DNA Stool, (b) E.Z.N.A. Stool DNA Kit, and (c) QIAmp Fast DNA Stool Mini Kit

	DNA yield		
	Ratio faecal slurry:water		
	1	1:1	1:9
NucleoSpin DNA Stool			
Fresh	141,040 ± 38,726	46,084 ± 14,581 (↓)	10,140 ± 7216 (↓)
Frozen	82,960 ± 10,844	30,228 ± 13,634 (↓)	12,652 ± 11,021 (↑)
E.Z.N.A. Stool DNA Kit			
Fresh	9188 ± 8363	2387 ± 2595 (↓)	176 ± 139 (↓)
Frozen	915 ± 883	284 ± 150 (↓)	6 ± 11 (↓)
QIAmp Fast DNA Stool Mini Kit			
Fresh	22 ± 22	6 ± 6 (↓)	0.3 ± 0.6 (↓)
Frozen	1079 ± 832	290 ± 388 (↓)	8 ± 12 (↓)

The arrows indicate how the expected DNA yield corresponds to the DNA yield in the undiluted sample

The price of DNA extraction (per sample) with the NS kit was estimated to be 2.77 €, whereas with the EZ and QA—2.20 € and 5.00 €, respectively.

## Discussion

Coproscopical methods based on microscopy for the quantification of GIN in grazing livestock are cumbersome and time-consuming as subsequent culturing of larvae is needed for genus identification. Nowadays, a variety of PCR-based applications can be employed to complement the identification, such as qPCR [20, 21], multiplex-tandem PCR [22] and ddPCR [16, 17]. All of these methodologies rely on the successful amplification of the internal transcribed spacer (ITS) regions located between the 18S and 5.8S subunits of the ribosome encoding genes [23]. Thus, a key step in any template amplification protocol is to obtain gDNA for subsequent molecular analyses, which is challenging when dealing with faecal material, due to the presence of various inhibitors and DNAses [24].

The application of various PCR-based tests in the diagnosis of GIN of veterinary importance has increased rapidly in the recent decade. Nevertheless there are only a few studies that compare how different DNA extraction protocols work on strongylid nematode eggs in grazing livestock [11, 20, 25]. To the best of our knowledge, however, none of these systematically compare the amount of DNA different commercial spin column extraction kits generate when applied directly to the eggs contained in faecal samples. This sharply contrasts with the situation for example with DNA extraction methods from bacterial samples [26–28]. Here we compared three different commercial DNA extraction kits in their capacities to produce consistent *Haemonchus* ITS-2 amplicon concentration measurements by utilizing the previously described ddPCR assay [16]. ITS-2 copy number concentrations were estimated in replicated, either fresh or frozen, faecal samples containing *Haemonchus* eggs, when prepared from a faecal slurry stock solution, as well as dilutions.

Although the DNA extracted herein was only analysed with our recently developed ddPCR platform, judging from the obtained ITS-2 amplicon copy number concentrations (Fig. 3) and the CV values at each sample dilution (Table 1), the overall best performing kit was NS. On top of being reasonably priced (2.77 € per sample), this spin-column based DNA extraction kit is based on a simple, easy to follow protocol, consisting of nine steps (in contrast to 14 for both EZ and QA). This makes it an attractive choice for the extraction of gDNA from strongyle eggs present in faecal material. The samples extracted with EZ generated overall lower amplicon concentration values (Fig. 3), and the obtained CV values for both fresh and previously frozen samples showed a greater degree of variation compared to NS, independent of dilution.

The lowest amplicon copy concentrations were obtained by using the QA, which in contrast to the previous two, relies solely on chemical lysis to disrupt the eggs. Although it has been shown that nematode eggs can also be cracked open by repeated freezing (− 80 °C) and heating cycles (105 °C) [20], our observations are confirmed by those of Harmon et al. [11], wherein strongyle egg disruption was best accomplished using a mechanical bead beating step. Furthermore, post freezing, most eggs were observed to be intact, which is also in agreement with Harmon et al. [11].

Having analysed the data shown in Fig. 3, we supposed that nematode eggs fall into three different states after having been frozen for 3 months: disrupted, partially disrupted or intact. Since freezing overall produced somewhat lower amplicon copy concentrations (in contrast to fresh samples), we assumed that a smaller portion of eggs are cracked open by freezing and that the gDNA degrades in this unstable environment. Another state the eggs can assume post-freezing, is that of being partially disrupted, i.e. having their shells partially cracked by freezing for an extended period, which would, in turn, help explain the higher amounts of gDNA recovered with the QA kit after sample storage at − 20 °C. However, the largest proportion of frozen eggs seem to stay intact, which subsequently explains the limited differences in amplicon copy concentration means observed within the NS and EZ kits, across dilutions.

It is important to acknowledge that the CV values generally increased with the increasing magnitude of dilution, which, in combination with the limited number of samples used herein, contributed to the decrease in power to determine truly significant differences (or lack thereof) between the different sample categories. In addition, storing the samples already dispensed and diluted in separate tubes before the freezing step (instead of a single stock solution, as was performed here) should also be addressed in future studies in order to examine if this alternative approach reduces the possible variation observed herein. Moreover, a systematic error in connection to diluted samples was observed, with the DNA yield for dilutions being lower than expected in comparison to the undiluted samples. The reason for this bias is unknown, but it can be argued that difficulties in connection to pipetting, including the use of cut pipette tips, may be the underlying cause. Alternative methods to facilitate the initial pipetting step should therefore be assessed.

In our study, inhibitors did not seem to play a major role, as the efficiency of the quantification declined as samples were diluted, which is unlike what has been observed in other similar studies, where diluting the samples seemed to improve both detection and quantification via decreases in the Ct values [15]. An inherent advantage of the ddPCR technology is also its capacity to monitor complex backgrounds for subtle changes in target amplicon levels [18], which cannot otherwise be detected with other real-time or qPCR assays, especially considering the presence of various inhibitors [19].

## Conclusions

We have found that the NS kit provides superior amounts of DNA extracted from *Haemonchus* eggs, leading to an overall more consistent quantification of the genus. Furthermore, even after 3 months of freezing, only a limited decline in amplicon DNA concentrations was observed. This presents a real possibility for the re-examination of the initially collected, screened (using traditional coproscopical methods) and stored samples, using molecular tools, such as ddPCR.

## Data Availability

The datasets used and/or analysed during the current study are available from the corresponding author on reasonable request.
